# Double Trouble: Hemophagocytic Lymphohistiocytosis in a Recent COVID-19 Infection Associated With Epstein-Barr Virus (EBV) Reactivation

**DOI:** 10.7759/cureus.82733

**Published:** 2025-04-21

**Authors:** Nicole Buleza, Ammaar Amanat, Nicole Terrigno

**Affiliations:** 1 Internal Medicine, Inspira Health Network, Mullica Hill, USA; 2 Internal Medicine, Cooper University Hospital, Camden, USA

**Keywords:** covid-19, ebv hlh, epstein-barr virus (ebv), hemophagocytic lymphohistiocytosis (hlh), infection-associated hlh

## Abstract

Hemophagocytic lymphohistiocytosis (HLH) is a rare, life-threatening syndrome of excessive immune activation triggered by infections, malignancy, or genetic abnormalities. While the Epstein-Barr virus (EBV) is commonly associated with HLH, COVID-19 still lacks a well-defined association with this syndrome. This case describes a woman with a recent COVID-19 infection with the presumed reactivation of EBV, leading to fatality secondary to HLH. Initial suspicion was high for acute cholecystitis. However, multiple repeat imaging was negative, and the patient's rapid decline and constellation of laboratory results and symptoms are highly suggestive of a secondary HLH due to EBV reactivation. Re-elevation of EBV viral capsid antigen immunoglobulin G (IgG) antibody can be an indicator of EBV reactivation, and COVID-19 patients with EBV reactivation appear to have an increased risk of death. This case represents a novel presentation of secondary HLH. Further studies are needed to establish the role of antiviral therapies, such as ganciclovir, in treatment as well as to evaluate the prognostic value of early antiviral administration in COVID-19-reactivated herpesvirus infections, such as EBV. However, whether antiviral therapy may decrease mortality in HLH triggered by COVID-19-reactivated EBV infections warrants further investigation.

## Introduction

Hemophagocytic lymphohistiocytosis (HLH) is a rare, life-threatening syndrome of excessive immune activation triggered by infections, malignancy, or genetic abnormalities. The Epstein-Barr virus (EBV) is a ubiquitous virus and is commonly associated with HLH [1,2]. COVID-19 however does not yet have a well-characterized association with this syndrome. Several studies indicate that severe COVID-19 infections can reactivate herpesviruses, particularly EBV [3,4]. Current literature review suggests that patients with recent or suspected EBV reactivation may have an increased risk of severe symptoms, including hemophagocytic syndromes [5]. The following describes the case of a woman with a recent COVID-19 infection with the presumed reactivation of EBV, complicated by fatality secondary to HLH.

## Case presentation

A 74-year-old woman with a history of atrial fibrillation, asthma on dupilumab, chronic obstructive pulmonary disease, and recent COVID-19 infection treated with nirmatrelvir/ritonavir presented to the emergency department for dyspnea and cough. Initial vital signs were notable for a fever of 38°C, tachycardia, hypoxia that improved on 2L nasal cannula, and hypotension. Physical exam was significant for mildly diminished breath sounds. Chest X-ray showed bibasilar atelectasis. Chest CT angiography was negative for pulmonary embolism.

**Figure 1:**
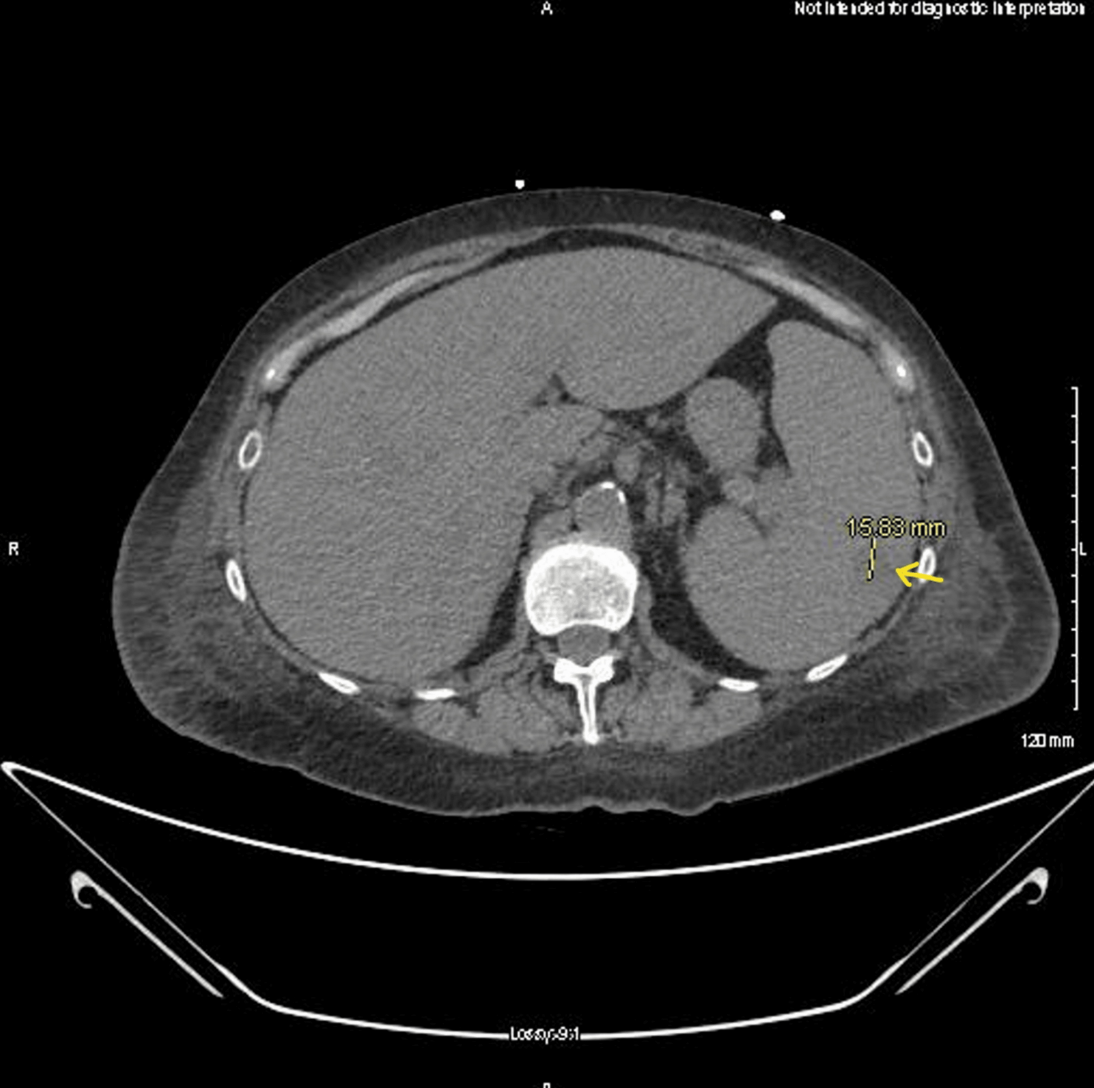
CT of the abdomen and pelvis: splenic hypoattenuating lesion with splenomegaly

**Figure 2:**
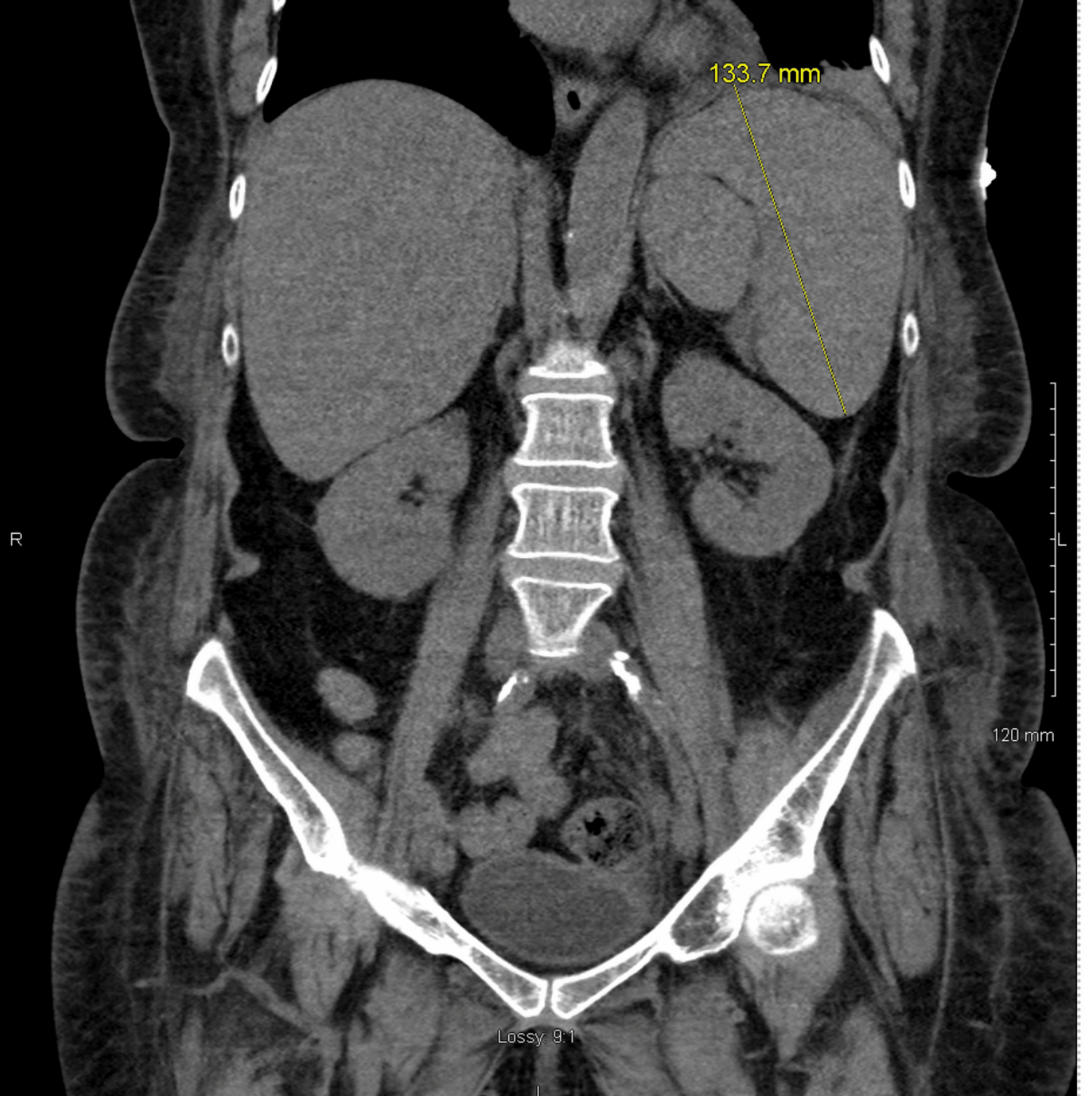
CT of the abdomen and pelvis: splenomegaly up to 13.4 cm (indicated with line). Calculated volume >900 mL

She was started on ceftriaxone, azithromycin, and guaifenesin for concerns of community-acquired pneumonia. She remained hypotensive despite adequate fluid resuscitation, and her antibiotic coverage was broadened. She acutely developed transaminase elevation and direct hyperbilirubinemia, with rising coagulation factors, and gastroenterology, infectious disease, and surgery were consulted. CT of the abdomen and pelvis was ordered and showed a contracted gallbladder with findings questionable for cholecystitis and splenomegaly with a splenic hypoattenuation, suspicious for a developing infarct (Figure 1 and Figure 2).

**Figure 3:**
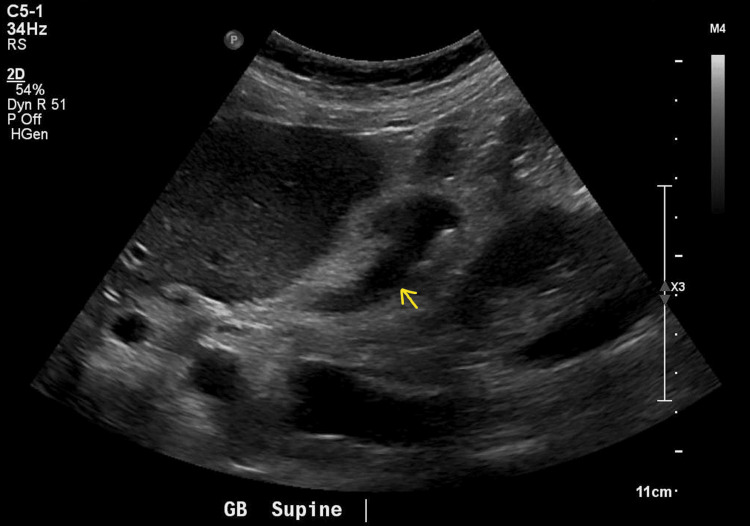
Abdominal ultrasound: contracted gallbladder with suspected cholelithiasis. No additional findings to suggest cholecystitis or biliary obstruction

A follow-up right upper quadrant ultrasound was ordered to evaluate for cholecystitis but again only showed a contracted gallbladder and suspected cholelithiasis (Figure 3).

**Figure 4:**
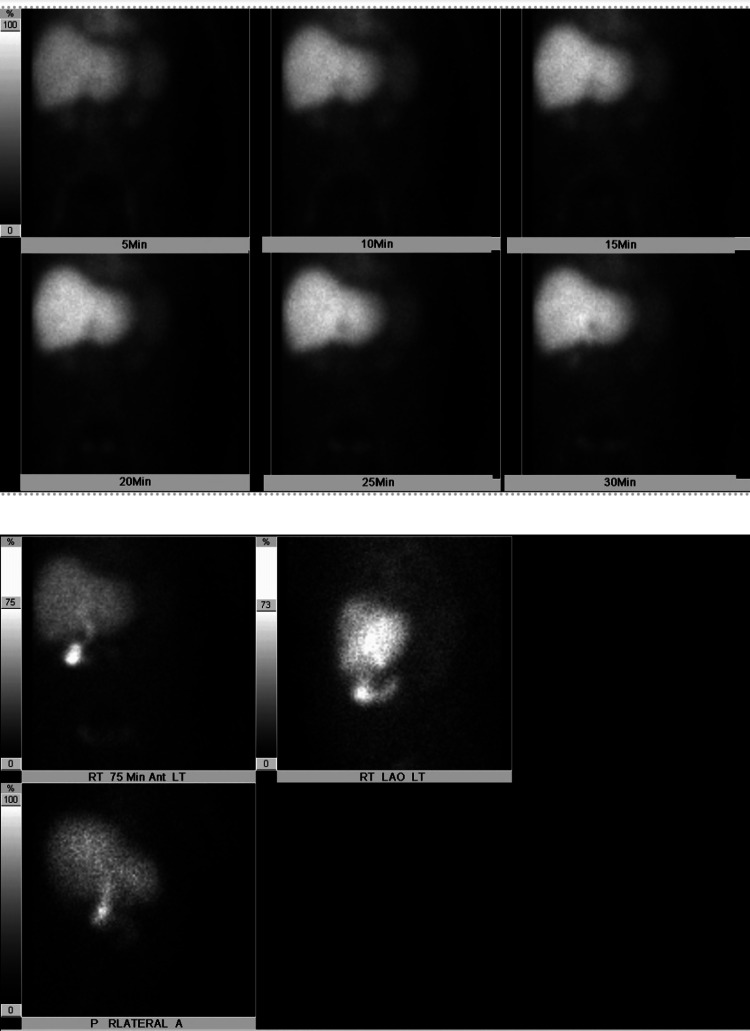
HIDA scan: non-visualized gallbladder. The common bile duct and duodenal loop and proximal jejunum are within normal limits HIDA: hepatobiliary iminodiacetic acid

**Figure 5:**
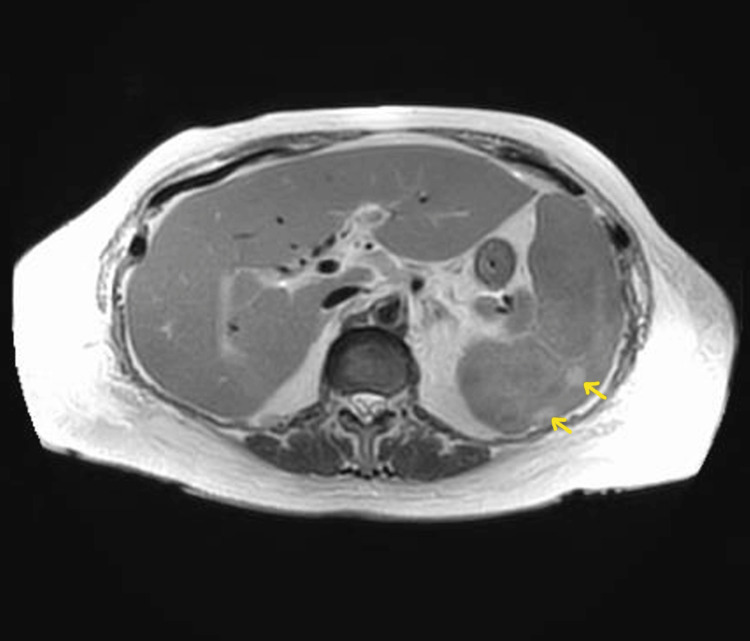
MRCP with interval increase in the size of the spleen with the development of numerous rounded lesions measuring up to 1.5 cm. Peripheral, wedge-shaped foci of T2 hyperattenuation within the spleen which may represent developing infarcts MRCP: magnetic resonance cholangiopancreatography

A hepatobiliary iminodiacetic acid (HIDA) scan resulted within normal limits. Magnetic resonance cholangiopancreatography (MRCP) was performed and showed an enlarged spleen with hyperattenuation, possibly representing a developing infarct and contracted gallbladder (Figure 4 and Figure 5).

Surgery recommended no surgical intervention. Hepatitis panel, herpes simplex virus (HSV), cytomegalovirus (CMV), human immunodeficiency virus (HIV), and EBV were ordered. On day 6, the patient's leukocytosis worsened to 16,000 cells/uL, and her lactic acid acutely increased from 2.2 to 6.2 mmol/L. She was transferred to the ICU for septic shock and was started on vasopressors and stress dose steroids with the suspected etiology being acute cholecystitis. However, repeat CT of the abdomen and pelvis was unchanged, again showing only contracted gallbladder. The viral panel results were as follows: The EBV viral capsid antigen immunoglobulin G (IgG) antibody level was elevated at >750 unit/mL. The EBV nuclear antigen antibody IgG was also high at >600 unit/mL. The EBV viral capsid antigen immunoglobulin M (IgM) antibody was normal. HIV, hepatitis, and CMV IgM were negative. Other lab work was notable for anemia, thrombocytopenia, low fibrinogen, elevated ferritin, high triglycerides, and worsening transaminase elevation. On day 9, the patient complained of severe abdominal pain, followed by respiratory failure requiring intubation. She had a stark increase in leukocytosis to 61,000 cells/uL (Table 1, Table 2, and Table 3).

**Table 1:** Complete blood count and lactic acid during hospitalization The bolded items are the values that fall outside the reference ranges for "normal". WBC: white blood cell; Hgb: hemoglobin; MCV: mean corpuscular volume

	Day 1	Day 2	Day 3	Day 4	Day 5	Day 6	Day 7	Day 8	Day 9	Reference
WBC	7.8	13.3	11.3	10.1	6.5	16.1	24.8	35.6	61.6	4.0-11.0 × 10^3^/uL
Hgb	12.9	12.0	10.7	11.2	11.0	11.9	10.7	11.0	9.5	11.0-15.2 g/dL
MCV	85.2	86.2	84.9	85.6	83.1	84.0	83.5	81.4	91.5	80.0-98.0 fL
Platelets	118	87	90	88	74	78	73	83	73	140-380 × 10^3^/uL
Lactic acid	1.7			2.2		6.2	6.9	4.1	4.8	0.5-2.2 mmol/L

**Table 2:** Viral and fungal serological test results The bolded items are the values that fall outside the reference ranges for "normal". Hep A IgM-Q: hepatitis A virus immunoglobulin M antibody, quantitative; Hep B IgM-Q: hepatitis B core antibody IgM, qualitative; Hep BS Ag-Q: hepatitis B surface antigen, quantitative; Hep C Ab-Q: hepatitis C virus antibody, qualitative; CMV Ab IgM-Q: cytomegalovirus antibody, IgM, qualitative; HIV 1 RNA Quant-Q: HIV-1 RNA quantification; EBV Nuc Ag EBNA Ab IgG-Q: Epstein-Barr virus nuclear antigen antibody IgG, qualitative; EBV Viral Ag VCA IgG-Q: Epstein-Barr virus viral capsid antigen, IgG, quantitative; EBV Viral Ag VCA IgM-Q: Epstein-Barr virus viral capsid antigen IgM, quantitative; SARS-CoV-2 (COVID-19): severe acute respiratory syndrome coronavirus 2 (COVID-19)

	Test result	Reference
*Aspergillus* antigen-Q	Not detected	Not detected
1,3-Beta-glucan	<31 pg/mL	Negative <31 pg/mL
Hep A IgM-Q	Non-reactive	Non-reactive
Hep B IgM-Q	Non-reactive	Non-reactive
Hep BS Ag-Q	Non-reactive	Non-reactive
Hep C Ab-Q	Non-reactive	Non-reactive
CMV Ab IgM-Q	<30.00 AU/mL	<30.00 AU/mL
HIV 1 RNA Quant-Q	Not detected	Not detected
EBV Nuc Ag EBNA Ab IgG-Q	>600.00 unit/mL	Positive >21.99 unit/mL
EBV Viral Ag VCA IgG-Q	>750.00 unit/mL	Positive >21.99 unit/mL
EBV Viral Ag VCA IgM-Q	36 unit/mL	Negative <36 unit/mL, equivocal 36-44 unit/mL, positive >44 unit/mL
SARS-CoV-2 (COVID-19)	Positive	Negative

**Table 3:** Coagulability workup and liver function testing The bolded items are the values that fall outside the reference ranges for "normal". PT: prothrombin time; PTT: partial thromboplastin time; INR: international normalized ratio; Alk Phos: alkaline phosphatase; ALT: alanine aminotransferase; AST: aspartate aminotransferase

	Day 1	Day 8	Day 9	Reference
PT	16.2	28.0		9.7-13.3 seconds
PTT		50.9		22.2-37.6 seconds
INR	1.5	2.5		0.8-1.2
Fibrinogen level		132		170-444 mg/dL
Ferritin		1567		16-288 ng/mL
Triglycerides		335		<150 mg/dL
Alk Phos	248		530	46-116 unit/L
ALT	24		62	10-49 unit/L
AST	68		353	0-34 unit/L
Total bilirubin	0.9		4.0	0.2-1.3 mg/dL
Direct bilirubin			3.2	0.0-0.3 mg/dL
Indirect bilirubin			0.8	0.0-0.1 mg/dL

A stat CT angiography of the abdomen and pelvis could not exclude bowel ischemia. The patient underwent emergent exploratory laparotomy, which was negative for bowel ischemia. She returned to the ICU on four vasopressors, unfortunately progressed to pulseless electrical activity (PEA) arrest, and ultimately passed away.

## Discussion

This patient met the criteria for HLH with fever, splenomegaly, hypertriglyceridemia, elevated ferritin, cytopenia, and low fibrinogen levels [2]. Unfortunately, her disease progressed rapidly and the diagnosis was made late. Early detection, diagnosis, and treatment of HLH are crucial, and while there may be therapeutic measures required to control the cytokine storm and suppress inflammation, the clinical courses are often poor and fulminant [1]. The etiology of this secondary HLH in the setting of a recent COVID-19 infection is likely due to EBV reactivation as other sources for her acute decline were negative including two repeat abdominal CT scans, negative right upper quadrant ultrasound, negative MRCP, an unchanged chest X-ray, and negative repeat blood cultures. During the window period of infection, EBV IgM can be within normal limits but IgG antibodies would remain elevated, as observed in this patient's serology. Furthermore, literature review notes that while EBV viral capsid antigen IgG can be a marker of previous infection, notable re-elevation of this marker also suggests EBV reactivation. This patient did not receive antiviral therapy. However, evidence from a retrospective study by Meng et al. suggests that ganciclovir may improve outcomes in similar cases. Among 217 COVID-19 patients with EBV serological testing, 55 (25.3%) demonstrated EBV reactivation defined by concurrent increases in EBV viral capsid antigen IgG and early antigen IgG. Of these patients with EBV reactivation, ganciclovir was found to have an increased 28-day survival rate compared to matched controls (p = 0.01) [3].

## Conclusions

Re-elevation of EBV viral capsid antigen IgG antibody can be an indicator of EBV reactivation. COVID-19 patients with EBV reactivation appear to have an increased risk of death, and further studies are needed to establish the role of antiviral therapies, such as ganciclovir, in treatment. This case demonstrates a novel presentation of secondary HLH. Additional studies are needed to evaluate the potential positive prognostic value of early antiviral administration in reactivated herpesvirus infections, such as EBV, in the context of COVID-19. However, the impact of antiviral therapies on mortality in HLH associated with COVID-19-reactivated EBV infections requires further investigation. 
